# The association between blood urea nitrogen to albumin ratio and the 28 day mortality in tuberculosis patients complicated by sepsis

**DOI:** 10.1038/s41598-024-65622-z

**Published:** 2024-07-16

**Authors:** Kunping Cui, Shuang Feng, Yi Mao, Haixia Luo, Jiao Yang, Ruyi Xu, Lang Bai

**Affiliations:** 1 Center of Infectious Diseases, West China Hospital of Sichuan University, Chengdu, 610041 Sichuan China; 2 Ultrasonic Medicine, Public Health Clinical Center of Chengdu, Chengdu, 610000 Sichuan China; 3 Intensive Care Unit, Public Health Clinical Center of Chengdu, Chengdu, 610000 Sichuan China

**Keywords:** Sepsis, TB, Blood urea nitrogen to albumin ratio, Prognosis, ICU, Infectious diseases, Risk factors

## Abstract

The relationship between blood urea nitrogen to albumin ratio (BAR) and the prognosis of patients with tuberculosis (TB) complicated by sepsis remains unclear. This study aimed to explore the association between BAR and overall patient prognosis. This was a retrospective cohort study of patients with TB complicated by sepsis who were admitted to the intensive care unit (ICU) of the Public Health Clinical Center of Chengdu between January 2019 and February 2023. The relationship between BAR values and prognosis in these patients was investigated using multivariate Cox regression, stratified analysis with interaction, restricted cubic spline (RCS), and threshold effect analysis. Sensitivity analyses were conducted to assess the robustness of the results. Our study included 537 TB patients complicated by sepsis admitted in the ICU, with a median age of 63.0 (48.0, 72.0) years; 76.7% of whom were men. The multivariate-restricted cubic spline analysis showed a non-linear association between BAR and patient prognosis. In the threshold analysis, we found that TB patients complicated by sepsis and a BAR < 7.916 mg/g had an adjusted hazard ratio (HR) for prognosis of 1.163 (95% CI 1.038–1.303; *P* = 0.009). However, when the BAR was ≥ 7.916 mg/g, there was no significant increase in the risk of death. The results of the sensitivity analysis were stable.

## Introduction

Tuberculosis (TB), a chronic infectious disease caused by *Mycobacterium tuberculosis*, remains a global public health concern. In 2022, there were 10.6 million new cases of TB worldwide, with 1.3 million deaths^1^. Despite significant progress in its prevention and treatment globally, some patients fail to receive a timely and accurate diagnosis as well as effective treatment during the early stages of the disease. This leads to disease progression, making patients susceptible to various other pathogenic infections and progressing to severe stages, such as sepsis, acute respiratory failure, and multiple organ dysfunction^2–4^. Sepsis is a severe organ dysfunction caused by a dysregulated body response to infection^5^. Each year, 50 million new cases of sepsis are reported, with approximately 10 million deaths and a mortality rate of 30%^6^. This has consistently been the focus of research in critical care medicine. The coexistence of TB and sepsis represents a convergence of the two severe diseases, creating substantial challenges for treatment and prognosis. Unfortunately, previous studies have often neglected this specific group of TB patients in the context of sepsis^7^. However, in recent years, scholars have begun to acknowledge this distinct population and have conducted further analyses, revealing that the mortality rate can increase to 40% among patients with TB complicated by sepsis^8,9^. Consequently, research exploring the prognosis of sepsis in patients with TB holds immense significance.

In clinical practice, blood biochemical indicators are commonly used to assess patient conditions and prognosis, with blood urea nitrogen (BUN) and albumin levels among the frequently tested parameters. The BUN level is an important indicator for renal function, water balance, and protein metabolism^10^. Albumin also plays a crucial role in maintaining colloid osmotic pressure and endothelial integrity as well as in regulating inflammation and immune function^11,12^. Previous studies have reported that elevated BUN and decreased albumin levels are closely associated with adverse outcomes in critically ill patients^13–15^. Several studies have validated the clinical significance of the blood urea nitrogen to albumin ratio (BAR) in predicting the prognosis of sepsis, severe pneumonia, and other diseases^16,17^.

Patients with TB (TB) complicated by sepsis experience severe systemic inflammatory response and nutritional depletion, leading to compromised kidney function and a significantly increased incidence of hypoalbuminemia. Thus, the BAR combines the nutritional and metabolic statuses of TB and sepsis, making it a potentially superior predictor of prognosis in patients with TB complicated by sepsis. However, current research has primarily focused on the association between blood urea nitrogen (BUN) or albumin levels and the prognosis of TB or sepsis^18,19^. There is a lack of in-depth research specifically on TB patients complicated by sepsis. Therefore, this study aimed to explore the correlation between the BAR and the prognosis of TB patients complicated by sepsis. The goal is to provide clinicians with a more accurate prognostic tool and treatment guidance, ultimately improving patient survival and quality of life.

## Patients and methods

### Study design and patients

**Figure 1:**
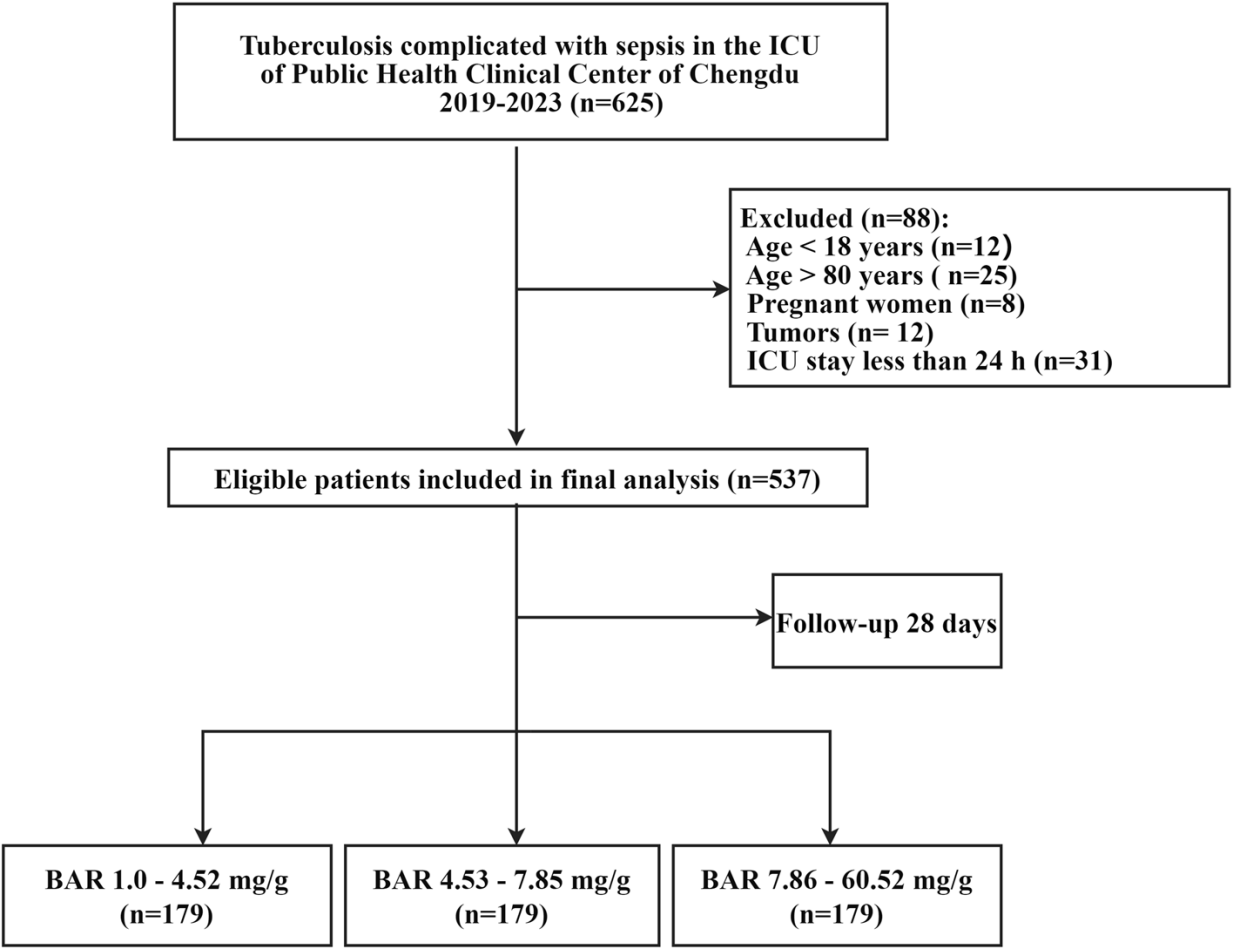
Flowchart of study patients included in this analysis.

This retrospective analysis included consecutive data from TB patients complicated by sepsis at a hospital specializing in infectious diseases (Public Health Clinical Center of Chengdu, Chengdu, Sichuan Province, China) between January 2019 and February 2023. Twelve patients younger than 18 years, 25 older than 80 years, eight pregnant women, 12 patients with tumors, and 31 patients who ICU stay less than 24 h were excluded from further analysis (Fig. 1). In reporting this study, we employed the Strengthening the Reporting of Observational Studies in Epidemiology (STROBE) guidelines^20^. The first and last authors conducted this study to ensure data accuracy and completeness.

### Definitions

TB was diagnosed based on CD10 expression^21^; clinical data were comprehensively analyzed to make a judgment^22^. Sepsis was defined as an infection combined with evidence of life-threatening organ dysfunction based on the third international consensus definitions^23^. Diagnosis of infection was based on the International Classification of Disease, Tenth Revision (ICD-10). Organ dysfunction was demonstrated by an increase of two or more points in the Sequential Organ Failure Assessment (SOFA) score^5,21^. The baseline SOFA score was assumed to be zero for all patients admitted in the ICU^24^. In addition, based on the patient's epidemiological data, clinical symptoms and auxiliary examination data, at least two critical care physicians made a diagnosis and treatment plan after discussion. BUN (1 mmol/L = 2.801 mg/dL) and albumin (1 g/L = 10 g/dL) levels were obtained from the first venous blood specimen within 24 h of the patient's admission to the ICU; BAR (mg/g) was calculated by dividing serum urea nitrogen (mg/dL) by serum albumin (g/dL).

### Variable extraction

Baseline clinical information was collected from electronic medical records within 24 h of admission to the ICU.

### Covariates

Based on published literature^7,16^ and clinical experience, the following covariates were included: demographic characteristics [i.e., sex, age, education level, body mass index (BMI), smoking, and alcohol abuse], comorbidities [i.e., chronic kidney disease (CKD), chronic liver disease, diabetes, hypertension, Acquired Immure Deficiency Syndrome (AIDS)], TB epidemiology (i.e., etiology of tuberculosis, anti-tuberculosis therapy, retreated TB, and drug-resistant TB), symptoms (fever, night sweats, dyspnea, and asthenia), laboratory test results [i.e., alanine aminotransferase (ALT), aspartate aminotransferase (AST), total bilirubin (TBIL), Blood Urea Nitrogen (BUN), albumin (ALB), creatinine, PaO2/FiO2 ratio (PF ratio), procalcitonin, and C-reactive protein], chest radiography (i.e., multilobar pulmonary infiltrates and cavitation), and interventions (i.e., mechanical ventilation use, vasopressor use, and renal replacement therapy).

Demographic information was obtained from the patients’ self-reports. Comorbidities were mostly revealed through patient self-reports or previous diagnoses. Educational level was divided into two levels based on education years (< 9 years and ≥ 9 years). BMI was calculated by dividing the weight (kg) by squared height (m^2^). Smoking was determined by answering the following question: “Have you smoked continuously or cumulatively for more than 6 months?”. Alcohol abuse was determined by answering the following question: “Have you consumed alcohol at least once a month for at least 6 months?”. The etiology of tuberculosis was based on phenotypic or molecular biological confirmation. The anti-tuberculosis therapy was divided into three groups: not initiation, intensive phase, and continuation phase. Retreated TB includes patients with failed initial treatment, patients whose sputum bacteria turn positive again after completing th regular anti-tuberculosis treatment, or patients who have been given irregular chemotherapy for more than one month. The diagnosis of drug-resistant TB was based on phenotypic or molecular biological confirmation; *Mycobacterium TB* was resistant to more than one anti-TB drug, along with a decision made after discussion between at least two senior TB specialists. Laboratory test results were obtained when the patients were admitted to the ICU. APACHE II and SOFA scores were calculated within 24 h from admission.

### Outcome ascertainment

The primary outcome of our study was death within 28 days of ICU admission. The study endpoint was determined upon collecting patient information.

### Statistical analysis

This study aimed to observe the association between the BAR and 28-day all-cause mortality in TB patients complicated by sepsis. Descriptive analysis was performed for all patients. The patients were divided into three groups based on the BAR tertiles. For continuous data, medians and interquartile ranges (IQR, quartiles 1–3) were used to express skewed distributions. Kruskal–Wallis tests were used for comparisons between groups. Categorical variables were expressed as proportions (%). The chi-square test or Fisher's exact test were used for comparison between groups.

To further analyze the independent association between BAR and 28-day all-cause mortality in TB patients complicated by sepsis, we used a multivariate Cox regression analysis; we showed (1) model 1 adjusted for sex and age, (2) model 2 adjusted for covariates from model 1 plus CKD, chronic liver disease, diabetes, hypertension, AIDS, etiology of tuberculosis, anti-tuberculosis therapy, retreated TB, and drug resistant TB, (3) model 3 adjusted for covariates from model 2 plus BMI, fever, ALT, AST, PaO2/FiO2 ratio, procalcitonin, C-reactive protein, mechanical ventilation use, and multilobar pulmonary infiltrates (*P*_*COX*_ < 0.1), (4) model 4 adjusted for variables from model 2 plus renal replacement therapy (effect value > 10%), and (5) model 5 adjusted for sex, age, BMI, CKD, chronic liver disease, diabetes, hypertension, AIDS, etiology of tuberculosis, anti-tuberculosis therapy, retreated TB, drug resistant TB, fever, ALT, AST, PaO2/FiO2 ratio, procalcitonin, C-reactive protein, mechanical ventilation use, renal replacement therapy, and multilobar pulmonary infiltrates, except BUN, ALB, APACHE II, and SOFA scores (based on collinearity factor consideration). The likelihood ratio test was used to assess the interactions across subgroups according to the respective subgroup indicators and age. Survival curves of the groups were plotted by Kaplan–Meier and log-rank analyses.

In addition, restricted cubic spline (RCS) regression was performed with four knots at the 5th, 35th, 65th, and 95th percentiles of BAR to investigate linearity as well as to examine the dose–response curve between BAR and 28-day all-cause mortality after adjusting for the variables in model 5. We also used a two-piecewise Cox regression model to examine the threshold association between age and 28-day all-cause mortality, adjusting for model 5 variables. A two-piece-wise Cox regression model was also used to analyze the association threshold between BAR and 28-day all-cause mortality after adjusting for the variables in model 5. A likelihood-ratio test was used to determine the inflection points. Finally, we excluded patients with AIDS, CKD and chronic liver disease from the sensitivity analyses to further evaluate the robustness of our results. All analyses were performed using R Statistical Software (Version 4.2.2, http://www.R-project.org, The R Foundation) and the Free Statistics analysis platform (Version 1.8, Beijing, China). A two-tailed test was performed; *P* < 0.05 was considered statistically significant.

### Ethics statement

This study followed the principles of the Declaration of Helsinki (revised in Brazil 2013). All experimental protocol were approved by the Ethics Review Board of the Public Health Clinical Center of Chengdu (Ethics number: YJ-K2022-16-01). Due to retrospective nature of the study need for informed consent of patients was Waived by the Research Ethics Committee of Public Health Clinical Center of Chengdu.

## Results

### Participant selection

In this study, 625 TB patients who developed sepsis were identified on the basis of the third international consensus definition. This study included 537 patients who were screened according to the inclusion and exclusion criteria. Figure 1 illustrates the flowchart for selecting study patients.

### Baseline characteristics

The baseline characteristics of the patients are presented in Table 1. The enrolled patients were divided into three groups by the tertiles of BAR as follows: Q1 group, 1.0–4.52 mg/g; Q2 group, 4.53–7.85 mg/g; and Q3 group, 7.86–60.52 mg/g. The median age of all patients was 63.0 (48.0, 72.0) years; 76.7% were men. There were some differences between the BAR groups with respect to various covariates (i.e., sex, age, alcohol abuse, CKD, diabetes, hypertension, fever, asthenia, AST, BUN, ALB, creatinine, procalcitonin, APACHE II score, SOFA score, and renal replacement therapy) (*P* < 0.05). Additionally, the overall 28-day all-cause mortality rate was 45.8%; the higher BAR group had a worse prognosis (*P* < 0.05).

**Table 1:** Characteristics of study patients by blood urea nitrogen to albumin ratio (BAR) tertiles.

Variables	Total	BAR (mg/g)			*P* value
		Q1 (1.0–4.52)	Q2 (4.53–7.85)	Q3 (7.86–60.52)	
No	537	179	179	179	
Patient characteristics					
Sex					0.002
Women	125 (23.3)	55 (30.7)	43 (24)	27 (15.1)	
Men	412 (76.7)	124 (69.3)	136 (76)	152 (84.9)	
Age (years)	63.0 (48.0, 72.0)	56.0 (41.0, 67.0)	65.0 (51.5, 73.5)	67.0 (54.0, 75.5)	< 0.001
Education level (years)					0.183
< 9	321 (59.8)	98 (54.7)	115 (64.2)	108 (60.3)	
≥ 9	216 (40.2)	81 (45.3)	64 (35.8)	71 (39.7)	
BMI (Kg/m2)	19.0 (18.3, 21.0)	19.3 (18.6, 21.7)	19.1 (18.3, 20.5)	19.0 (18.0, 20.3)	0.081
Smoking	299 (55.7)	86 (48)	98 (54.7)	115 (64.2)	0.008
Alcohol abuse	199 (37.1)	54 (30.2)	66 (36.9)	79 (44.1)	0.024
Comorbidities(%)					
CKD	34 (6.3)	0 (0)	3 (1.7)	31 (17.3)	< 0.001
Chronic liver disease	30 ( .6)	6 (3.4)	11 (6.1)	13 (7.3)	0.252
Diabetes	103 (19.2)	25 (14)	34 (19)	44 (24.6)	0.039
Hypertension	114 (21.2)	29 (16.2)	35 (19.6)	50 (27.9)	0.02
AIDS	17 (3.2)	5 (2.8)	6 (3.4)	6 (3.4)	0.941
TB epidemiology					
Etiology of tuberculosis					0.053
Negative	172 (32.0)	48 (26.8)	55 (30.7)	69 (38.5)	
Positive	365 (68.0)	131 (73.2)	124 (69.3)	110 (61.5)	
Anti-tuberculosis therapy					0.258
Not initiation	324 (60.3)	101 (56.4)	110 (61.5)	113 (63.1)	
Intensive phase	185 (34.5)	67 (37.4)	64 (35.8)	54 (30.2)	
Continuation phase	28 ( 5.2)	11 (6.1)	5 (2.8)	12 (6.7)	
Retreated TB					0.153
No	428 (79.7)	137 (76.5)	140 (78.2)	151 (84.4)	
Yes	109 (20.3)	42 (23.5)	39 (21.8)	28 (15.6)	
DR-TB	87 (16.2)	37 (20.7)	29 (16.2)	21 (11.7)	0.072
Symptoms (%)					
Fever	277 (51.6)	106 (59.2)	82 (45.8)	89 (49.7)	0.033
Night sweats	59 (11.0)	16 (8.9)	23 (12.8)	20 (11.2)	0.494
Dyspnea	474 (88.3)	163 (91.1)	158 (88.3)	153 (85.5)	0.26
Asthenia	188 (35.0)	50 (27.9)	58 (32.4)	80 (44.7)	0.003
Laboratory test results					
ALT (U/L)	23.0 (13.0, 42.0)	21.0 (12.0, 36.0)	24.0 (14.0, 43.0)	26.0 (12.0, 45.0)	0.331
AST (U/L)	35.0 (23.0, 65.0)	29.0 (20.0, 48.5)	40.0 (24.0, 68.0)	43.0 (25.5, 75.5)	< 0.001
TBIL (umol/L)	10.3 (6.9, 16.4)	9.7 (7.0, 14.4)	11.1 (6.8, 17.3)	10.3 (7.2, 17.4)	0.251
BUN (mg/dL)	16.3 (11.6, 25.4)	9.7 (7.9, 12.0)	16.3 (13.9, 19.0)	31.0 (24.2, 47.5)	< 0.001
ALB (g/dL)	2.8 (2.4, 3.1)	3.0 (2.7, 3.3)	2.8 (2.4, 3.2)	2.6 (2.2, 2.9)	< 0.001
Creatinine (umol/L)	57.0 (45.0, 81.7)	46.1 (38.1, 56.8)	57.2 (47.5, 74.1)	86.6 (57.0, 163.7)	< 0.001
PF ratio (mmHg)	162.7 (129.2, 193.9)	166.3 (136.0, 190.9)	165.0 (131.7, 189.4)	150.8 (117.6, 202.0)	0.498
Procalcitonin(ng/ml)	0.5 (0.2, 1.5)	0.2 (0.1, 0.8)	0.4 (0.2, 1.2)	1.1 (0.4, 4.5)	< 0.001
C-reactive protein(mg/L)	89.8 (51.0, 138.0)	82.4 (48.1, 124.4)	89.7 (49.4, 136.2)	97.3 (59.2, 151.3)	0.073
Chest radiography(%)					
Multilobar pulmonary infiltrates	340 (63.3)	109 (60.9)	112 (62.6)	119 (66.5)	0.531
Cavitation	37 ( 6.9)	12 (6.7)	15 (8.4)	10 (5.6)	0.576
Disease severity score					
APACH II score	17.0 (13.0, 21.0)	16.0 (13.0, 20.0)	16.0 (13.0, 20.0)	19.0 (16.0, 23.5)	< 0.001
SOFA score	4.0 (3.0, 6.0)	3.0 (3.0, 4.0)	4.0 (3.0, 6.0)	6.0 (4.0, 8.0)	< 0.001
Interventions (%, boolean for 1st 24 h)					
Mechanical ventilation use	158 (29.4)	45 (25.1)	54 (30.2)	59 (33)	0.258
Vasopressor use	191 (35.6)	56 (31.3)	64 (35.8)	71 (39.7)	0.253
Renal replacement therapy^a^	11 ( 2.0)	0 (0)	0 (0)	11 (6.1)	< 0.001
Outcome					
28-day mortality (Death)	246 (45.8)	45 (25.1)	84 (46.9)	117 (65.4)	< 0.001

Data are presented as median (IQR) or N (%).

*BAR* blood urea nitrogen-to-albumin ratio, *IQR* interquartile range, *BMI* body mass index, *CKD* chronic kidney disease, *AIDS* Acquired Immure Deficiency Syndrome, *DR-TB* drug-resistant TB, *ALT* alanine aminotransferase, *AST* aspartate aminotransferase, *TBIL* total bilirubin, *BUN* Blood Urea Nitrogen, *ALB* albumin, *PF ratio* PaO_2_/FiO_2_ ratio, *APACHE II* Acute Physiology and Chronic Health Evaluation, *SOFA* Sequential Organ Failure Assessment.

^a^Fisher's exact test.

### Outcomes

**Figure 2:**
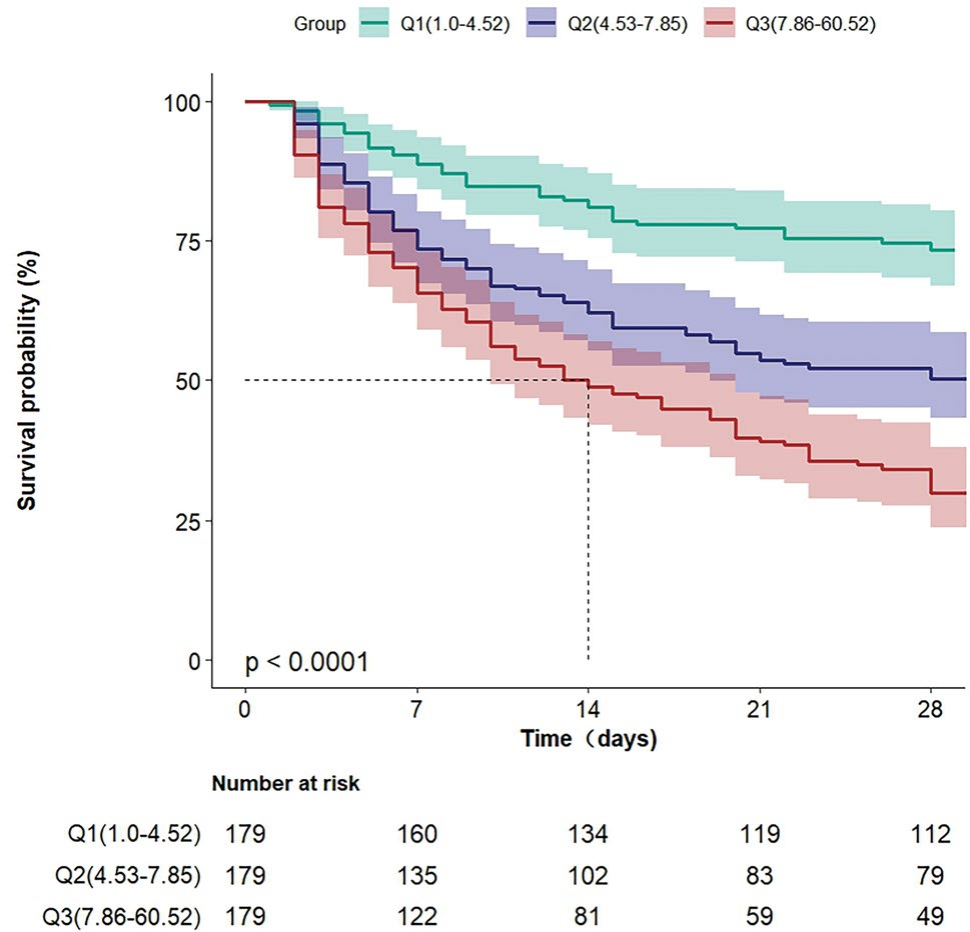
Kaplan–Meier survival analysis of different BAR groups in TB patients complicated by sepsis.

The overall 28-day all-cause mortality was 45.8%. Table 1 shows the 28-day all-cause mortality rates in different BAR groups. The 28-day all-cause mortality rates in groups 1–3 were 25.1%, 46.9%, and 65.4%, respectively. Kaplan–Meier curves showed that the higher the BAR, the higher the risk of 28-day all-cause mortality (log-rank test: *P* = 0.0001, Fig. 2).

### BAR and 28-day all-cause mortality

The results of the multivariate COX regression models are shown in Table 2, which were used to assess the relationship between the BAR and 28-day all-cause mortality in TB patients complicated by sepsis, are shown in Table 2. In the continuous model 1 adjusted for sex and age, the BAR was positively associated with 28-day all-cause mortality (hazard ratio [HR], 1.04; 95% confidence interval [CI], 1.02–1.05; *P* < 0.001). Despite adjusting for various covariates, the risk of 28-day all-cause mortality increased by 4% for each 1 mg/g increase in BAR. At the same time, in the categorical models 1 to 5, which were also adjusted for covariates (Table 2), the categorized BAR in the multivariate COX regression model seemed to confirm a linear relationship between BAR and 28-day all-cause mortality. The 1.0–4.52 mg/g BAR group had the lowest 28-day all-cause mortality rate.

**Table 2:** Relationship between BAR and the 28-day all-cause mortality in TB patients complicated by sepsis.

Exposure	Model 1		Model 2		model 3		Model 4		Model 5	
	HR (95% CI)	*P* value	HR (95% CI)	*P* value	HR (95% CI)	*P* value	HR (95% CI)	*P* value	HR (95% CI)	*P* value
BAR (mg/g)	1.04 (1.02–1.05)	< 0.001	1.04 (1.02–1.05)	< 0.001	1.04 (1.02–1.05)	< 0.001	1.04 (1.02–1.05)	< 0.001	1.04 (1.02–1.06)	< 0.001
BAR tertiles (mg/g)										
Q1(1.0–4.52)	1 (Ref)		1 (Ref)		1 (Ref)		1 (Ref)		1 (Ref)	
Q2(4.53–7.85)	2.14 (1.49–3.09)	< 0.001	2.2 (1.53–3.18)	< 0.001	1.97 (1.36–2.84)	< 0.001	2.2 (1.53–3.18)	< 0.001	1.97 (1.36–2.84)	< 0.001
Q3(7.86–60.52)	3.45 (2.43–4.9)	< 0.001	3.58 (2.49–5.16)	< 0.001	3.02 (2.07–4.4)	< 0.001	3.59 (2.49–5.16)	< 0.001	3.01 (2.06–4.39)	< 0.001
* P* for trend		< 0.001		< 0.001		< 0.001		< 0.001		< 0.001

Model 1 adjusted for sex and age.

Model 2 adjusted for covariates from model 1 plus CKD, chronic liver disease, diabetes, hypertension, AIDS, etiology of tuberculosis, anti-tuberculosis therapy, retreated TB, and drug resistant TB.

Model 3 adjusted for covariates from model 2 plus BMI, fever, ALT, AST, PaO2/FiO2 ratio, procalcitonin, C-reactive protein, mechanical ventilation use, and multilobar pulmonary infiltrates (PCOX < 0.1).

Model 4 adjusted for variables from model 2 plus renal replacement therapy (effect value > 10%).

Model 5 adjusted for sex, age, BMI, CKD, chronic liver disease, diabetes, hypertension, AIDS, etiology of tuberculosis, anti-tuberculosis therapy, retreated TB, drug resistant TB, fever, ALT, AST, PaO_2_/FiO_2_ ratio, procalcitonin, C-reactive protein, mechanical ventilation use, renal replacement therapy, and multilobar pulmonary infiltrates.

*HR* hazard ratio.

### Subgroup analyses

**Figure 3:**
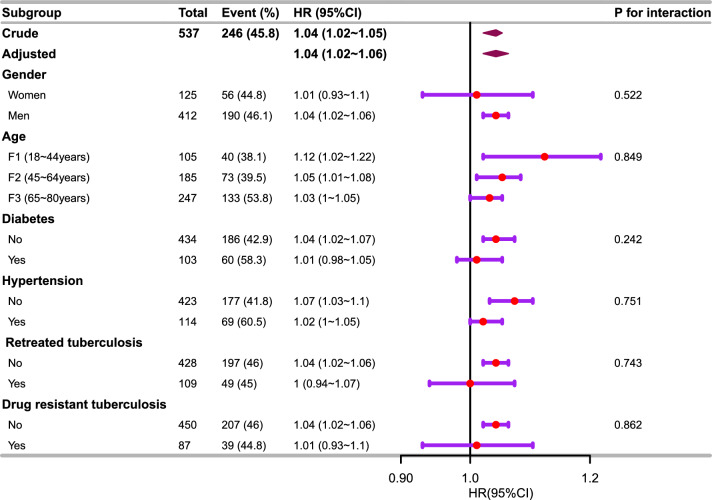
The relationship between BAR and the 28-day all-cause mortality in the subgroup analysis based on TB patients complicated by sepsis. Adjusted for sex, age, BMI, CKD, chronic liver disease, diabetes, hypertension, AIDS, etiology of tuberculosis, anti-tuberculosis therapy, retreated TB, drug resistant TB, fever, ALT, AST, PaO_2_/FiO_2_ ratio, procalcitonin, C-reactive protein, mechanical ventilation use, renal replacement therapy, and multilobar pulmonary infiltrates. HR, hazard ratio; CI, confidence interval.

The results of the subgroup analyses are shown in Fig. 3. BAR was associated with 28-day all-cause mortality among men (HR 1.04; 95% CI 1.02–1.06), those aged 18–44 years (HR 1.12; 95% CI 1.02–1.22), those aged 45–64 years (HR 1.05; 95% CI 1.01–1.08), those without diabetes (HR 1.04; 95% CI 1.02–1.22), these without hypertension (HR 1.07, 95% CI 1.03–1.1), non-retreated TB (HR 1.04; 95% CI 1.02–1.06), and those without drug-resistant TB (HR 1.04; 95% CI 1.02–1.06). There was no association among women, patients aged 65–80 years, diabetes, hypertension, retreated TB, and drug-resistant TB.

### Non-linear relationship between BAR and 28-day all-cause mortality

**Figure 4:**
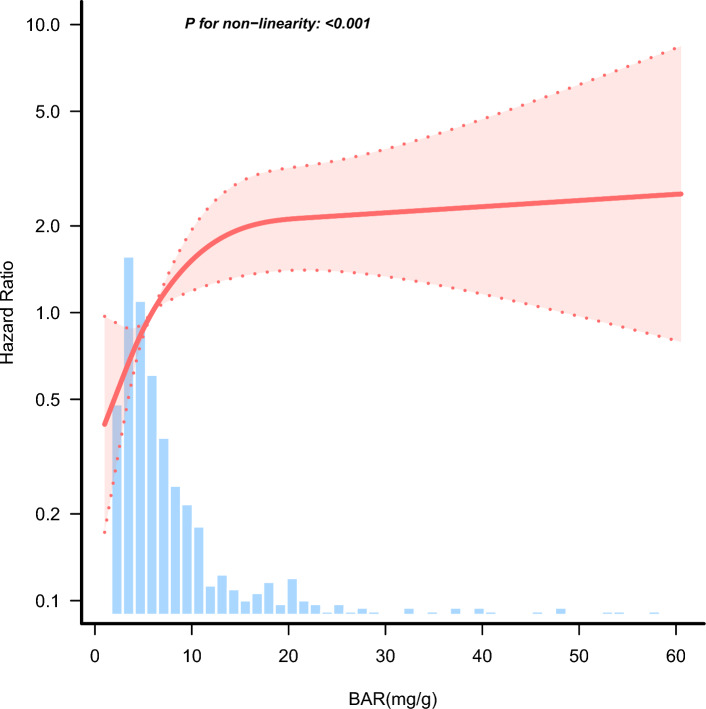
Association between BAR and the hazard ratio of 28-day all-cause mortality in TB patients complicated by sepsis. Solid and dashed lines represent the predicted value and 95% confidence intervals. They were adjusted for sex, age, BMI, CKD, chronic liver disease, diabetes, hypertension, AIDS, etiology of tuberculosis, anti-tuberculosis therapy, retreated TB, drug resistant TB, fever, ALT, AST, PaO2/FiO2 ratio, procalcitonin, C-reactive protein, mechanical ventilation use, renal replacement therapy, and multilobar pulmonary infiltrates. All of the data is displayed.

After adjusting for sex, age, BMI, CKD, chronic liver disease, diabetes, hypertension, AIDS, etiology of tuberculosis, anti-tuberculosis therapy, retreated TB, drug resistant TB, fever, ALT, AST, PaO_2_/FiO_2_ ratio, procalcitonin, C-reactive protein, mechanical ventilation use, renal replacement therapy, and multilobar pulmonary infiltrates, we observed a non-linear relationship between BAR and 28-day all-cause through a restricted cubic spline (*P* for non-linearity < 0.001, Fig. 4). Using a two-piecewise Cox regression model adjusted for sex, age, BMI, CKD, chronic liver disease, diabetes, hypertension, AIDS, etiology of tuberculosis, anti-tuberculosis therapy, retreated TB, drug resistant TB, fever, ALT, AST, PaO_2_/FiO_2_ ratio, procalcitonin, C-reactive protein, mechanical ventilation use, renal replacement therapy, and multilobar pulmonary infiltrates, we found that the BAR threshold was 7.916 mg/g (Table 3). Below the threshold, the 28-day all-cause mortality rose rapidly (HR 1.163; 95% CI 1.038–1.303; *P* = 0.009; Table 3); above the threshold, the 28-day all-cause mortality did not rise rapidly, wherein the estimated dose–response curve appeared to be within a consistent horizontal line (HR 1.036; 95% CI 0.997–1.075; *P* = 0.069; Table 3). This suggests that when the BAR was below 7.916 mg/g, the risk of 28-day all-cause mortality increased by 16.3% per 1 mg/g increase in BAR. In contrast, when the BAR value was greater than 7.916 mg/g, the risk of 28-day all-cause mortality increased by only 3.6% for every 1 mg/g increase in BAR.

**Table 3:** Threshold effect analysis of the relationship of BAR and the 28-day all-cause mortality in TB patients complicated by sepsis.

BAR (mg/g)	No.	Adjusted model	
		HR(95%CI)	P-value
< 7.916	361	1.163 (1.038, 1.303)	0.009
≥ 7.916	176	1.036 (0.997, 1.0075)	0.069
Likelihood Ratio test		–	0.01

Adjusted for sex, age, BMI, CKD, chronic liver disease, diabetes, hypertension, AIDS, etiology of tuberculosis, anti-tuberculosis therapy, retreated TB, drug resistant TB, fever, ALT, AST, PaO_2_/FiO_2_ ratio, procalcitonin, C-reactive protein, mechanical ventilation use, renal replacement therapy, and multilobar pulmonary infiltrates. 99% of the data are displayed.

*HR* hazard ratio, *CI* confidence interval.

### Sensitivity analyses

Although we excluded patients with comorbidities, such as AIDS, CKD, chronic liver disease, or both CKD and chronic liver disease, the multivariate COX regression model, after adjusting for each covariate, still demonstrated a 2% to 5% increase in the 28-day all-cause mortality for each 1-mg/g increase in BAR (Table 4). Additionally, our analysis using restricted cubic splines revealed a non-linear relationship between BAR and 28-day all-cause mortality in TB patients complicated by sepsis in the ICU (All *P* values for non-linearity < 0.05).

**Table 4:** Sensitivity analyses.

Analysis	Unweighted patients/total patients, No.	Adusted HR (95%CI)^a^	*P* value
Excluding AIDS	241/520	1.02 (1.01~1.04)	*P* = 0.001
Excluding CKD	223/503	1.05 (1.03~1.07)	*P* < 0.001
Excluding chronic liver disease	237/507	1.03 (1.02~1.05)	*P* < 0.001
Excluding CKD and chronic liver disease	215/474	1.05 (1.03~1.07)	*P* < 0.001

*HR* Hazard ratio, *CI* confidence interval, *CKD* chronic kidney disease.

^a^Adjusted for sex, age, BMI, diabetes, hypertension, etiology of tuberculosis, anti-tuberculosis therapy, retreated TB, drug resistant TB, fever, ALT, AST, PaO_2_/FiO_2_ ratio, procalcitonin, C-reactive protein, mechanical ventilation use, and multilobar pulmonary infiltrates.

## Discussion

In this retrospective cohort study, BAR was associated with 28-day all-cause mortality in TB patients complicated by sepsis among adjusted models. After excluding AIDS, CKD, chronic liver disease, or both CKD and chronic liver disease, due to the higher baseline urea nitrogen in CKD and lower baseline albumin in chronic liver disease, a sensitivity analysis was conducted with the multivariate COX regression model and restricted cubic spline; we adjusted for covariates and remained the association. Specifically, as the BAR level increased, the risk of mortality gradually increased. However, once the BAR exceeds 7.916 mg/g, the risk of mortality began to exhibit a linear trend. This indicated that the inflammatory response and severe organ dysfunction reached a certain limit or entered a chronic phase; after which, further increases in BAR levels no longer had a significant impact on prognosis.

A previous meta-analysis demonstrated a positive association between BAR and poor prognosis among patients with pneumonia^25^. Two meta-analyses focusing on COVID-19 confirmed this conclusion^26,27^. To the best of our knowledge, there are only three studies^16,28,29^ investigating the association between BAR and sepsis. One study^16^ confirmed that BAR can serve as an important indicator for the prognostic evaluation of patients with sepsis in the ICU. Another study^28^ conducted in 2022, which utilized data from the Medical Information Market for Intensive Care IV Sample, reported an adjusted HR of 1.266 (95% CI 1.126–2.3) for the association between high- and low-BAR groups in sepsis patients. The adjustment factors included 16 variables: age, SOFA score, and anion gap. Furthermore, another study^29^ found a positive association between the high-BAR group and 30-day mortality in sepsis patients, with an HR of 1.219 (95% CI 1.095–1.357) after controlling for age, systolic blood pressure, and diastolic blood pressure. While previous studies on sepsis did not include patients with TB, our findings confirmed that the BAR may be a significant feasibility indicator for assessing the prognosis of TB patients complicated by sepsis.

The development and progression of TB and sepsis encompass intricate acute and chronic evolutionary processes. The factors influencing the prognosis of TB patients complicated by sepsis remain complex. Our research showed that there was a close correlation between the BAR, a combined indicator of BUN and ALB, and the prognosis of TB patients complicated by sepsis. Although theoretically, BAR might be affected by patients' baseline nutritional status, our research data indicate that the median levels of BMI, BUN, and ALB for all patients were 19.0 (18.3, 21.0) kg/m^2^, 16.3 (11.6, 25.4) mg/dL, and 2.8 (2.4, 3.1) g/dL, respectively. Among them, patients with higher BAR levels were more likely to have higher BUN and lower ALB contentcompared with patients in the lowest BAR tertile group. However, no statistically significant difference was observed in terms of BMI. This finding indicates that nutritional status did not significantly impact the BAR ratio, further emphasizing the crucial clinical value of BAR in evaluating patient prognosis. BAR may increase the risk of death in these patients via multiple intrinsic mechanisms. When patients experience severe infections and inflammation, their bodies enter a state of high metabolism, which can lead to protein breakdown for energy supply. This can result in the elevation of BUN while simultaneously reducing albumin synthesis, thus leading to decreased albumin levels. Malnutrition and the inflammatory state weaken the immune function of the body, thus increasing the risk for complications and death^30–32^. Second, severe infections and inflammatory responses can cause kidney injury, resulting in elevated BUN and disruption of the internal environment, thereby adversely affecting the body's recovery and regulatory abilities^33–35^. Thirdly, elevated BUN and decreased albumin levels may reflect excessive release of mediators, cytokine abnormalities, and impaired immune system, thus increasing the risk of death in patients^36–39^.

Our study demonstrated that BAR has significant clinical value in the prognostic assessment of TB patients complicated by sepsis, and can be used as a simple and effective tool for physicians to identify high-risk patients at an early stage as well as to develop individualized treatment strategies. However, this study had several limitations. First, because of the retrospective nature of the study, we could not determine the temporal association between TB and sepsis. Additional well-designed cohort studies are required to confirm these findings. Second, as with all observational studies, there may have been some uncontrolled potential confounding factors. Although we conducted adjustments for covariates and performed sensitivity analyses to validate the strong association between BAR and overall prognosis, it is important to note that unknown confounding factors and unmeasured variables may still exist. Therefore, further confirmation from prospective, large-sample studies may be required to validate these findings. Third, our study population was only from a single center, which inevitably led to selection bias. The results of this study should be further verified by conducting multi-center, prospective, cohort studies.

## Conclusions

The findings of this retrospective cohort study suggest a non-linear association between BAR and prognosis among TB patients complicated by sepsis. Some complications of sepsis may result in renal impairment and decreased albumin levels during the exacerbation phase of the disease; therefore, our findings may be important for clinicians to monitor disease changes and provide timely treatment.

## Data Availability

The data supporting the findings of this study are available from the first author, Kunping Cui, upon request.
